# Case report: From misdiagnosis to timely detection: a clinical and imaging guide to neurological presentations of diffuse large B-cell lymphoma—insights from six cases

**DOI:** 10.3389/fonc.2025.1410953

**Published:** 2025-02-10

**Authors:** Chunxiao Yang, Zihua Gong, Tao Wang, Huijuan Yuan, Weinan Na, Wei Xie, Shengyuan Yu

**Affiliations:** ^1^ Department of Neurology, The Second Medical Center, Chinese People’s Liberation Army (PLA) General Hospital, Beijing, China; ^2^ Departm ent of Neurology, Bethune International Peace Hospital, Shijiazhuang, Hebei, China; ^3^ Department of Critical Care Medicine, The Fourth Medical Center, Chinese People’s Liberation Army (PLA) General Hospital, Beijing, China; ^4^ School of Medicine, Nankai University, Tianjin, China; ^5^ Department of Neurology, The First Medical Center, Chinese People's Liberation Army (PLA) General Hospital, Beijing, China

**Keywords:** DLBCL, PCNSL, neurological symptoms, misdiagnosis, case report

## Abstract

**Background:**

The clinical spectrum of diffuse large B-cell lymphoma (DLBCL) is notably heterogeneous. Some DLBCL patients initially present with neurological manifestations, leading to their preliminary diagnosis within neurology departments. However, the overlap of clinical and auxiliary examination findings with those of various neurological entities—such as cerebral infarction, demyelination, viral encephalitis, and peripheral neuropathy—often results in diagnostic misattribution.

**Case presentation:**

We delineate six pathologically-confirmed DLBCL cases, each heralded by neurological deficits, including limb paresis, sensory loss, vertigo, seizure activity, and aphasia. These presentations precipitated multiple erroneous diagnoses pertaining to nervous system pathologies, culminating in a median diagnostic latency of 8 months.

**Conclusion:**

The differential diagnostic process for the misdiagnosed conditions in these cases has been meticulously revisited, enhancing the diagnostic acumen of neurologists. These cases underscore the imperative for neurologists to maintain a high index of suspicion for lymphoma in atypical presentations and to judiciously integrate multimodal diagnostic modalities—such as comprehensive imaging, cerebrospinal fluid analysis, and biopsy—to expedite diagnosis and initiate timely intervention.

## 1 Introduction

Diffuse large B-cell lymphoma (DLBCL) is the most common type of non-Hodgkin’s lymphoma (NHL) in adults, accounting for 35%–50% of NHL cases in China. DLBCL typically presents as rapidly enlarging lymph nodes, and most patients are diagnosed and treated by hematologists. However, DLBCL can also involve any tissue or organ as the primary site, resulting in diverse clinical manifestations (1). Some patients with DLBCL initially present with neurological symptoms and are first seen by neurologists. Their clinical features and ancillary tests can mimic various neurological disorders, such as stroke, demyelination, viral encephalitis, and peripheral neuropathy. Therefore, neurologists should be familiar with DLBCL and its diagnosis to avoid treatment delay. We report six cases of DLBCL confirmed by biopsy, all of which had neurological symptoms as the first presentation, including four cases of primary central nervous system lymphoma (PCNSL) and two cases of secondary central nervous system lymphoma (SCNSL). We analyzed their clinical and radiological characteristics to improve the differential diagnosis skills of neurologists for lymphoma.

## 2 Case presentation

A comprehensive summary of the clinical and diagnostic features for all six patients is presented in **Table 1**. Notably, none of the patients had HIV infection or a history of malignancy, autoimmune disease, or organ transplantation.

**Table 1:** Clinical and diagnostic features of six DLBCL patients with neurological symptoms.

	P1	P2	P3	P4	P5	P6
Gender	F	F	F	M	F	F
Age (y)	54	26	52	55	65	49
Location of the lesion	Brain	Brain	Brain	Brain	Cauda equina, paranasal sinus, and adrenal gland	Spinal cord, cauda equina, paraspinal soft tissues, and callosum
Onset pattern	Acute	Acute	Subacute	Acute	Chronic	Chronic
Initial symptoms	Limb weakness	Seizure	Dizziness	Motor aphasia	Limb numbness	Limb numbness
Peak symptoms	Headache and diplopia	Cognitive impairment	Limb weakness and oculomotor paralysis	Epilepsy	Limb weakness	Bowel and bladder dysfunction
Misdiagnosis	Cerebral infarction and nonspecific inflammation	Viral encephalitis and acute disseminated encephalomyelitis	Brainstem encephalitis and multiple sclerosis	Viral encephalitis	Peripheral neuropathy and spinal tuberculosis	Nonspecific inflammation
Time to diagnosis (M)	2.5	9	10	2.5	7	12

### 2.1 Patient 1

A 54-year-old female presented with sudden onset of left-sided numbness and weakness. Initial MRI suggested a right midbrain and thalamus infarction, with high signals on diffusion-weighted imaging (DWI); however, her symptoms did not improve with treatment. A few days later, she developed headaches and diplopia. CT revealed mixed low-density lesions in the right thalamus, midbrain, pons, and medulla oblongata. MRI showed low T1, high T2, and high DWI signal lesions with patchy enhancement in the right basal ganglia, thalamus, midbrain, and pons. Cerebrospinal fluid (CSF) analysis revealed elevated protein levels (830.3 mg/L) with normal glucose, chloride, and leukocyte counts. The diagnosis was “non-specific inflammation”, and her symptoms significantly improved after corticosteroid and immunosuppressive therapy, with a reduction in lesion size. Two months later, her symptoms recurred and progressively worsened. Follow-up MRI findings are detailed in **Table 2**. A biopsy of the right thalamus was performed, revealing DLBCL, positive for CD20, PAX5, BCL-2, BCL-6, and MUM1, and negative for CD10, indicating a non-germinal center B-cell-like (non-GCB) subtype. Postoperatively, the patient received rituximab, high-dose methotrexate, and cytarabine. She had no significant medical or family history.

**Table 2:** Latest imaging findings of six DLBCL patients with neurological symptoms.

Patient ID	Imaging Modality	Regions	Findings	Impression
P1	CT	Right thalamus, midbrain, pons, and medulla oblongata	Low-density	–
	MRI	Right basal ganglia, thalamus, midbrain, and pons	Slightly low T1, high T2, high DWI signal, patchy enhancement; Mild mass effect, perilesional edema; MRS and ASL (right basal ganglia): elevated Cho and normal NAA peak; hypoperfusion	Possible nonspecific inflammation
P2	CT	Right frontal lobe, basal ganglia and thalamus	Low-density	–
	MRI	Bilateral frontal lobe, parietal lobe, temporal lobe, insular cortex, cerebral peduncle, hippocampus, periventricular regions, basal ganglia, thalamus, cerebellar hemisphere and callosum	Low T1, high T2, slightly high DWI signal, homogeneous enhancement (right temporal lobe); No mass effect, no perilesional edema	Possible primary intracranial tumor
P3	CT	Bilateral basal ganglia, thalamus, midbrain, pons, and medulla oblongata	Low-density	
	MRI	Bilateral frontal lobe, periventricular regions, basal ganglia, thalamus, midbrain, pons, medulla oblongata and callosum	Slightly low T1, high T2, iso/high DWI signal, patchy/homogeneous enhancement; Mass effect, perilesional edema; MRS (right basal ganglia): elevated Cho, decreased NAA, and presence of Lip peak; ASL: normal perfusion	Possible neoplastic lesions (e.g., lymphoma)
	FDG-PET/CT	Left frontal lobe, bilateral basal ganglia, right thalamus, pons, medulla oblongata, and cerebellum	Increased localized FDG uptake	Possible neoplastic lesions (e.g., lymphoma)
P4	CT	Bilateral frontal lobe	Low-density	
	MRI	Bilateral frontal lobe	Slightly low T1, high T2, high DWI signal, homogeneous enhancement; No mass effect, no perilesional edema; MRS and ASL (right frontal lobe): elevated Cho and normal NAA peak; hyperperfusion	Possible neoplastic lesions (e.g., lymphoma) or inflammatory lesions
	FDG-PET/CT	Left frontal lobe	Increased localized FDG uptake	Possible neoplastic lesions (e.g., lymphoma)
P5	MRI	Spinal pia mater and cauda equina	Irregular spinal cord margins, iso/slightly high T1, iso/slightly high T2, heterogeneous enhancement	Possible infectious lesions
	FDG-PET/CT	Paranasal sinuses, bilateral margins of the T1 vertebra, L2-L4 spinal canal, adrenal glands, right atrial appendage, and bilateral nipples	Increased localized FDG uptake	Possible neoplastic lesions (e.g., lymphoma)
P6	HRCT	Bilateral L2 to S3 nerve roots and left L5 nerve root	Enlargement and thickening	–
	MRI	Splenium of the corpus callosum, spinal cord and dural matter at the T11–L1 level, cauda equina, and L4 paraspinal soft tissues	Slightly low T1, high T2, high DWI signal, heterogeneous enhancement	Possible nonspecific inflammation
	FDG-PET/CT	Whole-body	Normal FDG uptake	–

### 2.2 Patient 2

A 26-year-old female presented with recurrent seizures. CT showed low-density lesions in the right frontal lobe and basal ganglia. MRI revealed low T1, high T2, and slightly high DWI signals in the right frontal lobe, parietal lobe, hippocampus, bilateral insular cortex, periventricular regions, and basal ganglia, with faint patchy enhancement in the right insular cortex. CSF analysis was normal. Initially diagnosed with “viral encephalitis”, she was treated with antiviral and antiepileptic therapy. However, she experienced progressive cognitive decline. Five months later, follow-up MRI indicated lesion expansion, and CSF analysis showed positive oligoclonal bands. The diagnosis was revised to “acute disseminated encephalomyelitis (ADEM)”, and corticosteroid therapy improved her symptoms and reduced lesion size. Post-discharge, her cognitive impairment worsened, with increased sleepiness and difficulty walking. Follow-up MRI findings are detailed in **Table 2**. A biopsy of the right temporal lobe confirmed DLBCL, positive for CD20, PAX5, BCL-2, BCL-6, and CD10, indicating a GCB subtype. Postoperatively, she received chemotherapy with rituximab and high-dose methotrexate. The patient is a hepatitis B virus carrier with no other significant medical or family history.

### 2.3 Patient 3

**Figure 1:**
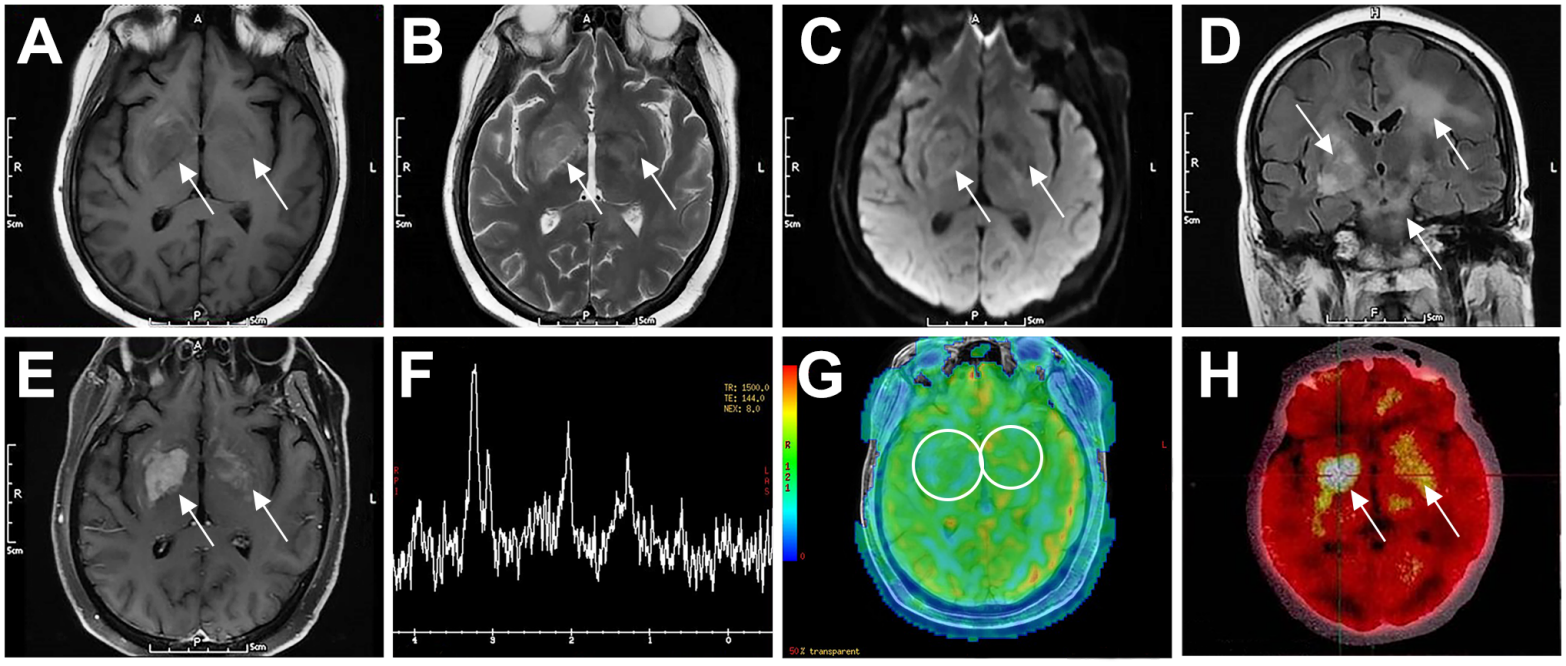
Typical imaging features of a 52-year-old female patient with PCNSL (P3). The patient had bilateral basal ganglia lesions (right larger than left), which showed low signal on T1WI **(A)**, high signal on T2WI **(B)**, slightly high signal on DWI **(C)**, and diffuse high signal on T2-Flair **(D)**. The lesions showed inhomogeneous enhancement on contrast-enhanced MRI **(E)**. MRS showed decreased NAA peak (2.02 ppm), increased Cho peak (3.20 ppm) and Lip peak (0.9–1.3 ppm) in the right basal ganglia lesion **(F)**. ASL perfusion imaging showed no abnormal hyperperfusion **(G)**. FDG-PET/CT showed focal increased radioactivity uptake in both basal ganglia, more prominent on the right side, with SUVmax of 21.7 **(H)**. Arrows indicate lesions. CNS, central nervous system; DLBCL, diffuse large B-cell lymphoma; T1WI, T1-weighted image; T2WI, T2-weighted image; DWI, diffusion-weighted imaging; Flair, fluid-attenuated inversion recovery; MRS, magnetic resonance spectroscopy; NAA, N-acetyl-aspartate; Cho, choline; Lip, lipid; ASL, arterial spin labeling; FDG-PET/CT, fluorodeoxyglucose-positron emission tomography/computed tomography; SUV, standard uptake value.

A 52-year-old female presented with dizziness for two months. Diagnosed with “functional vestibular disorder” and treated with circulatory improvement therapy, her symptoms did not improve. She then developed left-sided weakness and diplopia. MRI revealed abnormal signals with enhancement in the right thalamus, medulla, pons, cerebral peduncle, and periaqueductal gray matter. Repeated CSF analysis showed mildly elevated protein levels, with cytology revealing a few lymphocytes but no malignant cells. She was subsequently diagnosed with “brainstem encephalitis” and “multiple sclerosis”, and received two courses of corticosteroid therapy combined with intravenous immunoglobulin or plasma exchange, along with immunosuppressive drugs. Her symptoms partially improved with each course but progressively worsened overall. Seven months later, CSF protein levels elevated to 1175.5 mg/L, while other indicators remained normal, and no malignant cells were found in CSF cytology. Blood and CSF tests were negative for AQP4-Ab, NMO-IgG, and oligoclonal bands. Latest imaging findings are detailed in **Table 2** and illustrated in **Figure 1**. A biopsy of the right basal ganglia confirmed DLBCL, positive for CD20, PAX5, BCL-2, BCL-6, and CD10, indicating a GCB subtype. Postoperatively, she received chemotherapy with rituximab and high-dose methotrexate. Her medical history included hyperlipidemia, with no other significant medical or family history.

### 2.4 Patient 4

A 55-year-old male presented with sudden motor aphasia. MRI revealed mixed signals in the left frontal lobe with significant enhancement and surrounding edema. FDG-PET/CT indicated increased localized FDG uptake in the left frontal lobe. He received mannitol for dehydration therapy, and one month later, follow-up MRI showed a reduction in the lesion size. Initially diagnosed with “viral encephalitis”, he was treated with acyclovir, resulting in symptom improvement. However, one month after discharge, the patient experienced a sudden seizure. Follow-up MRI findings are detailed in **Table 2**. CSF analysis was normal. A biopsy of the right frontal lobe was performed, revealing DLBCL, positive for CD20, PAX5, and MUM1, with BCL-2, BCL-6, and CD10 negative, indicating a non-GCB subtype. The patient received chemotherapy with rituximab, high-dose methotrexate, and cytarabine. His medical history included surgeries for gallstones and cataracts, and his father had died of esophageal cancer, with no other significant medical or family history.

### 2.5 Patient 5

A 65-year-old female presented with progressive limb numbness for over three months. Electromyography indicated peripheral neuropathy. She was treated with neurotrophic drugs, corticosteroids, and mannitol, but symptoms persisted. Subsequently, she developed progressive limb weakness and a persistent low-grade fever. Blood tests showed a positive Tb-spot test, and CSF analysis revealed mildly elevated leukocyte counts and protein levels, suggesting a possible CNS infection, potentially spinal tuberculosis. Despite anti-tuberculosis treatment and corticosteroids, her condition worsened. Four months later, repeat CSF analysis showed leukocyte counts of 48×10^6^/L, glucose at 0.8 mmol/L, chloride at 112.8 mmol/L, and protein at 1810.5 mg/L. CSF cytology revealed numerous lymphocytes but no malignant cells. Imaging findings are detailed in **Table 2**. A biopsy of the right nipple confirmed DLBCL, positive for CD20, PAX5, BCL-2, BCL-6, and MUM1, and negative for CD10, indicating a non-GCB subtype. She received a combination of rituximab and lenalidomide for treatment. She experienced a weight loss of approximately 5 kg over a year, with no other significant medical or family history.

### 2.6 Patient 6

**Figure 2:**
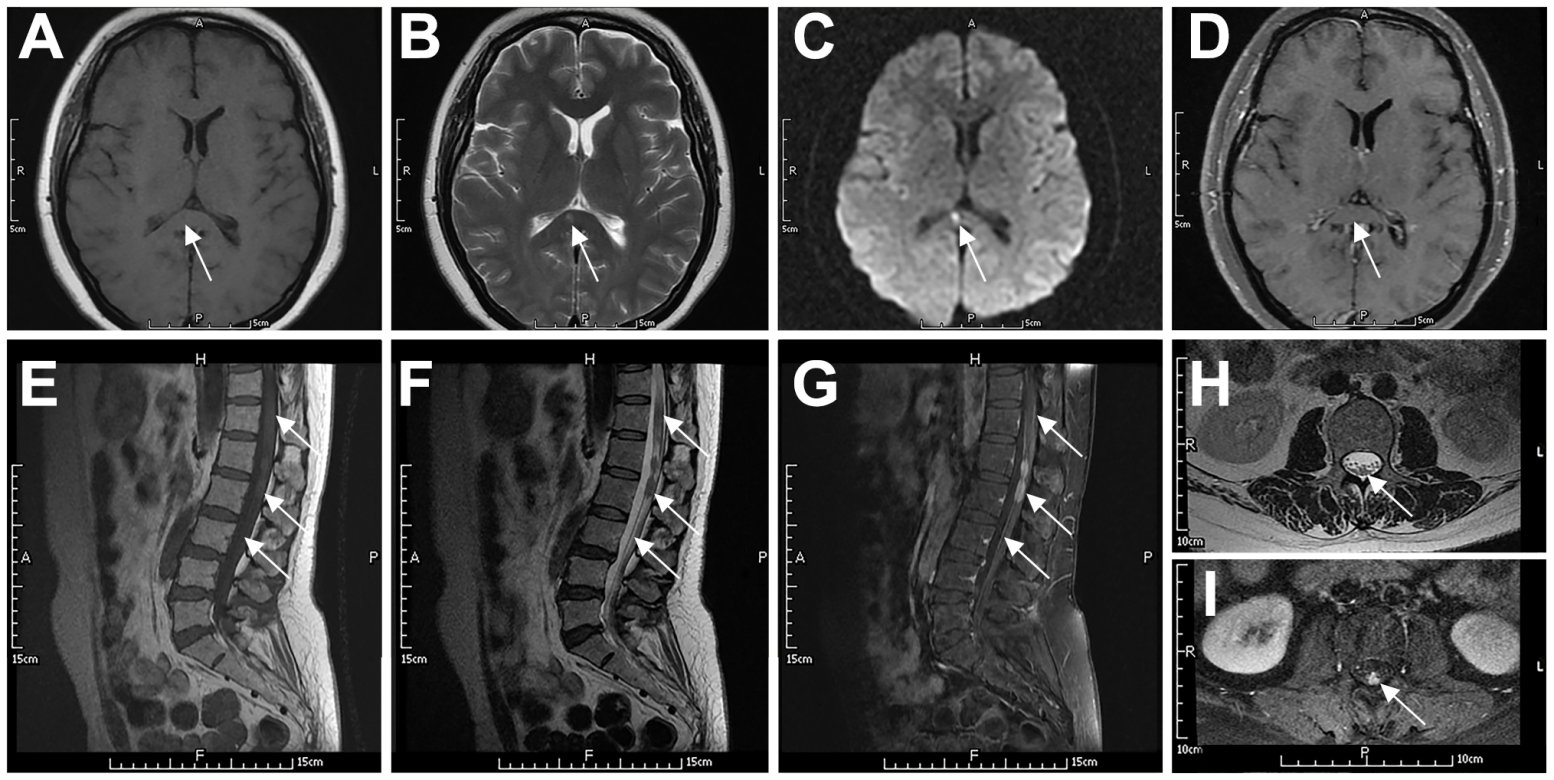
Typical imaging features of a 49-year-old woman with systemic DLBCL (P6). The patient had slightly prolonged T1 **(A)** and T2 **(B)** signals in the splenium of the corpus callosum, with a high DWI signal **(C)** and no contrast enhancement **(D)**. The spinal cord and cauda equina at the T11–L2 level showed patchy or nodular T2 **(F, H)** and T1 **(E)** signals. The spinal cord had mild enhancement, the local pia mater had abnormal enhancement **(G)**, and the cauda equina had nodular enhancement **(I)**. Arrows indicate lesions.

A 49-year-old female presented with progressive numbness and weakness in both lower limbs for six months, which worsened with bowel and bladder dysfunction. CSF protein was 690 mg/L. MRI of the thoracolumbar spine showed suspected thickening of the posterior dura at the T12-L1 level. Whole-body FDG-PET/CT showed Normal FDG uptake. Suspecting “non-specific inflammation”, she was treated with corticosteroids and intravenous immunoglobulin, resulting in significant symptom improvement. Five months later, her symptoms recurred. Repeat CSF analysis showed a pressure of 235/190 mmHg, glucose at 2.4 mmol/L, chloride at 120.2 mmol/L, protein at 1267 mg/L, and a leukocyte count of 12×10^6^/L. Oligoclonal bands in CSF were positive, while those in serum were negative. The PPD test was negative. MRI findings are detailed in **Table 2** and illustrated in **Figure 2**. A cauda equina biopsy confirmed DLBCL, positive for CD20, PAX5, BCL-2, and BCL-6, and negative for CD10, indicating a non-GCB subtype. She was treated with a combination of rituximab and lenalidomide. Her medical history included hypertension, deep vein thrombosis of the left lower limb, and a hysterectomy, with no other significant medical or family history.

## 3 Discussion

### 3.1 Clinical and imaging findings

DLBCL is a highly invasive type of NHL, with an increasing incidence projected since 2020 (2). The median age at diagnosis is in the mid-60s, with 30% of patients being older than 75 years. The clinical manifestations of DLBCL are highly heterogeneous, with major symptoms and signs varying based on the affected regions of the nervous system. For instance, the bilateral basal ganglia and right midbrain lesions in Patient 3 resulted in limb weakness and oculomotor paralysis, while the conus medullaris and cauda equina lesions in Patient 6 led to limb numbness and bowel and bladder dysfunction.

As listed in **Table 3**, the imaging characteristics of PCNSL can be considered typical, including supratentorial involvement, with lesions frequently located in the basal ganglia, periventricular regions, midline, and corpus callosum, consistent with our cases (P1-P4). Lymphoma lesions typically appear hyperdense on CT and hypointense on MRI T2 sequences, with marked diffusion restriction on DWI, reflecting the high density of lymphoma cells. These lesions exhibit either heterogeneous or homogeneous enhancement, often accompanied by leptomeningeal, subependymal, and perivascular enhancement patterns. Generally, this tumor shows low-to-intermediate cerebral blood volume. In FDG-PET/CT, most lesions exhibit high FDG avidity with homogeneous uptake (3). The imaging findings of our patients are largely consistent with these typical features, except that the CT scans of our four patients showed hypodensity, and the T2 sequences were slightly prolonged, potentially due to prior corticosteroid therapy, resulting in atypical imaging features. Additionally, Patient 6 exhibited normal FDG uptake, which may be due to the long duration of his disease, the slow growth of the tumor cells, and the involvement of the dura mater, which typically shows lower metabolic activity.

**Table 3:** Key clinical and radiographic differences between PCNSL, TDLs, and herpes simplex encephalitis.

	PCNSL in Immunocompetent Patients	TDLs	Herpes Simplex Encephalitis
Onset	Acute/subacute/chronic	Acute/subacute	Acute/subacute
Symptoms	Focal neurological symptoms	Preceding infections or vaccinations, focal neurological symptoms	Prodromal symptoms, fever, focal neurological symptoms
Location of the lesions	Midline structures (basal ganglia, periventricular regions, and corpus callosum)	White matter	Limbic system, medial temporal lobe, insular cortex, and inferolateral frontal lobe
CT	Hyperdense (without corticosteroid)	Hypodense	Normal/early hypodense, later hyperdense (hemorrhage)
MRI	Low T2, high DWI, low ADC; homogeneous enhancement; mass effect, perilesional edema; low-to-intermediate perfusion; Cho peak↑, NAA peak↓, Lip peak	High T2, high DWI, high ADC; open or closed ring-enhancement; No mass effect, mild perilesional edema; hypoperfusion; Cho peak↑, NAA peak↓	High T2, high DWI, low ADC; variable enhancement pattern; cerebral edema; hyperperfusion; Cho peak↑, NAA peak↓
FDG-PET/CT	High FDG uptake	Low to moderate FDG uptake	Hypermetabolism
CSF Analysis	Mild protein elevation; IL10↑, IL6↑; positive CSF cytology and flow cytometry	Mild protein elevation; related autoimmune markers	Moderate protein, lymphocyte, and erythrocyte elevation; positive HSV PCR

The symbol “↑” represents an increase, while “↓” indicates a decrease.

### 3.2 Differential diagnosis

**Figure 3:**
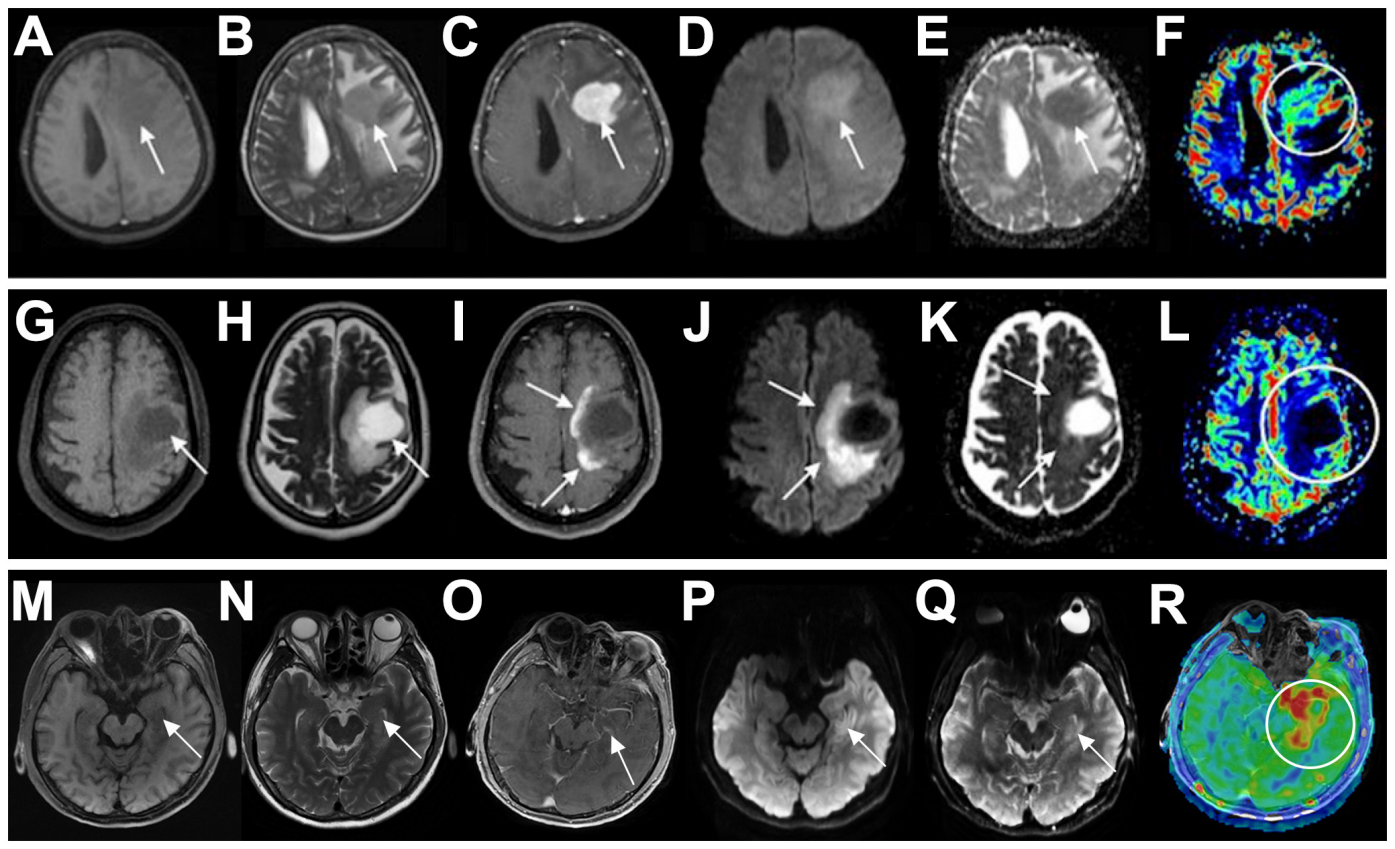
Differential diagnosis of PCNSL in immunocompetent patients. **(A-F)** A 71-year-old woman with PCNSL. The patient presented with a solid expansive lesion in the left frontal white matter, characterized by low signal on T1 **(A)** and T2 **(B)**, with homogeneous contrast enhancement **(C)**. The lesion exhibited high signal on DWI **(D)** and low signal on the ADC map **(E)**. rCBV imaging showed mild hyperperfusion [circle in **(F)**]. **(G–L)** A 41-year-old woman with TDLs. The patient presented with an expansive lesion in the left subcortical frontal white matter, showing low signal on T1 **(G)** and high signal on T2 **(H)**, accompanied by perilesional edema and incomplete ring-enhancement **(I)**. The lesion also demonstrated incomplete peripheral restricted diffusion on DWI **(J)** and ADC maps **(K)** and no signs of hyperperfusion on rCBV imaging [circle in **(L)**]. **(M–R)** A 58-year-old man with herpes simplex encephalitis. The left medial temporal lobe was swollen, exhibiting slightly low signal on T1 **(M)** and high signal on T2 **(N)**, with no significant contrast enhancement **(O)**. The lesion presented slightly restricted diffusion on DWI **(P)** and ADC maps **(Q)** and hyperperfusion on rCBF imaging [circle in **(R)**]. Arrows indicate lesions. PCNSL, primary central nervous system lymphoma; DWI, diffusion-weighted imaging; ADC, apparent diffusion coefficient; rCBV, relative cerebral blood volume; TDLs, tumefactive demyelinating lesions; rCBF, relative cerebral blood flow.

Lymphoma is a great mimicker with a challenging differential diagnosis. PCNSL is very challenging to differentiate from demyelinating diseases, especially in young immunocompetent patients. Demyelinating diseases often have an acute or subacute onset, with a relapsing course, and often have a history of infection or vaccination before onset, but the clinical presentation is nonspecific. Immune markers can be positive in CSF. On imaging, tumefactive demyelinating lesions (TDLs) is the most difficult to distinguish from PCNSL. The features of TDLs include ring enhancement, iso- and high-mixed signals on T2, and the absence of cortical involvement and mass effect (4). PCNSL showed strong diffusion restriction, whereas TDLs showed increased apparent diffusion coefficient (ADC). Although acute demyelinating lesions may have peripheral diffusion restriction corresponding to ring enhancement areas, the ADC of TDLs is not as low as that of PCNSL (5). The specific clinical and radiographic differences between PCNSL and TDLs are detailed in **Table 3** (6, 7) and illustrated in **Figures 3A–L** (8).

The differentiation between PCNSL and viral encephalitis (mainly herpes simplex encephalitis) is relatively simple. The latter has an acute onset, often with fever and prodromal symptoms. A key differential feature on MRI is the presence of hemorrhage in herpes simplex encephalitis, which causes mixed signal changes on T1- and T2-weighted images, whereas PCNSL is usually nonhemorrhagic. Moreover, the serum virus antibody titer of herpes simplex encephalitis is markedly elevated, the CSF virus PCR test is positive, the disease course is self-limited, and antiviral therapy is effective. As the disease progresses, viral encephalitis can be quickly excluded in patients with PCNSL. Pierre Giglio et al. reported a case of PCNSL mimicking the clinical and MRI features of herpes simplex encephalitis. The patient was diagnosed by biopsy after antiviral treatment failed to improve the symptoms and images (9). The specific clinical and radiographic differences between PCNSL and herpes simplex encephalitis are detailed in **Table 3** (10–12) and illustrated in **Figures 3A−F, M−R**).

**Figure 4:**
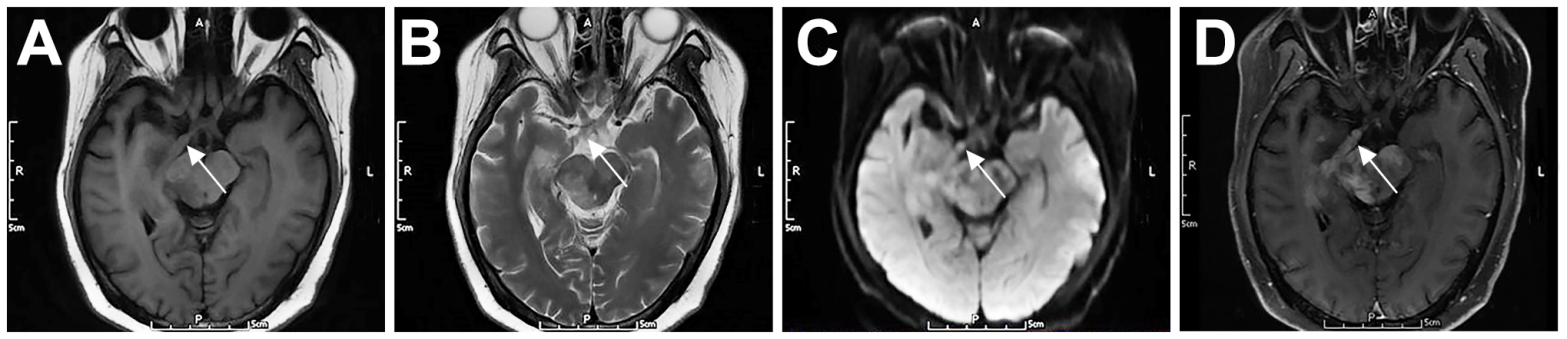
Cranial nerve involvement in a 52-year-old female patient with PCNSL (P3). The right optic tract was enlarged, with iso T1 **(A)** and iso T2 **(B)** signals, slightly high DWI signal **(C)**, and marked contrast enhancement **(D)**. Arrows indicate lesions.

Approximately 5% of DLBCL patients may develop cranial neuropathy (13). This condition, also known as neurolymphomatosis (NL), occurs when lymphoma cells infiltrate the cranial or peripheral nerves, nerve roots, or plexuses (14). The clinical diagnosis of NL is challenging, and the number of reported cases is low; therefore, the prevalence of the disease remains unclear (15). In this series, patient 3 was diagnosed with PCNSL by right basal ganglia biopsy. Cranial MRI (**Figure 4**) showed enlargement of the right optic tract, with equal T1 and T2 signals, slightly high DWI signal, and marked contrast enhancement. Although the optic tract was not biopsied, lymphoma involvement was inferred from the patient’s symptoms and imaging findings.

NL is more difficult to diagnose when it occurs isolated or predominantly in the lumbosacral nerve root and cauda equina. CSF analysis helps to exclude other diseases, such as infection, but it has little value for lymphoma diagnosis. Electrophysiological testing is useful to locate the lesion and guide further imaging studies. Imaging is the most valuable tool for diagnosing NL. Marquardt et al. reported four cases of lymphoma with lumbosacral radiculopathy as the initial presentation. All patients had abnormal enhancement on MRI, including two cases of cauda equina and two cases of extraspinal lumbosacral plexus (16). FDG-PET/CT scans can assist in the diagnosis of NL with a sensitivity of 84%–91%, which may be helpful when MRI is negative (17). However, the MRI findings are not specific for NL, and similar findings can be observed in patients with neurofibroma, schwannoma, sarcoidosis, or infection. Patient 5 was misdiagnosed with spinal tuberculosis due to overlapping clinical and imaging features. Both conditions can present with similar symptoms such as back pain, fever, weight loss, and neurological deficits. Imaging studies like MRI may show similar findings, such as spinal lesions and soft tissue masses (18, 19). Therefore, MRI results should be interpreted in conjunction with clinical and laboratory findings. Timely biopsies of the affected areas shown by MRI or FDG-PET/CT scans are essential for a definitive diagnosis.

### 3.3 Lessons learned

When encountering neurological symptoms of unknown origin, to avoid delays in the diagnosis of DLBCL, we recommend first gathering detailed information about the patient’s symptoms and medical history. Comprehensive imaging, including CT, MRI, or FDG-PET/CT (if necessary), should be performed as soon as possible to identify any masses or abnormalities and to assess the extent of the disease. If symptoms progress, repeated imaging should be conducted immediately. Dynamic clinical and imaging observations are crucial for the diagnosis of lymphoma. A lumbar puncture can be performed to aid in diagnosis. Notably, CSF cytology is the most commonly utilized technique for diagnosing leptomeningeal dissemination of lymphoma. When cytology results are negative, immunophenotyping via flow cytometry can be beneficial. However, a review of 1,481 immunocompetent patients with PCNSL revealed that only a minor fraction of patients could be diagnosed through CSF analysis, with detection rates ranging from 0% to 4% of cases (20). Au Ka Loong Kelvin and colleagues (21) indicated that CSF analysis is more likely to yield positive results in PCNSL patients with a history of hematologic malignancy or abnormal MRI enhancement. Consequently, the standard diagnostic approach for PCNSL remains timely brain biopsy whenever feasible. CSF cytology combined with flow cytometry should be employed only when there is no accessible mass for biopsy, highly suspected lymphoma, or when the mass is located in a high-risk surgical area. Among our six patients, only two underwent CSF cytology, both of which yielded negative results; ultimately, all six patients were diagnosed via biopsy. Before the diagnosis is determined, corticosteroid therapy should be used with caution. Additionally, it is crucial to consult a multidisciplinary team to review the case and ensure all aspects are considered. This approach facilitates prompt and accurate diagnosis, leading to timely and effective treatment.

## 4 Conclusion

DLBCL presenting with neurological symptoms as the first manifestation is prone to misdiagnosis. When these patients initially presented to our neurology department, the average time to diagnosis extended to seven months. During this period, they were frequently misdiagnosed and even received corticosteroid treatment, which significantly complicated the diagnostic process and adversely affected their prognosis. This study investigates the clinical and imaging findings of six DLBCL patients, and discusses the differential diagnosis of the disorders that were initially mistaken for lymphoma, aiming to highlight the complexities involved in the diagnostic and treatment processes and to broaden the clinical perspective of neurologists. For patients with suspected lymphoma, comprehensive imaging, CSF analysis, and timely biopsy should be performed to achieve an early diagnosis and prompt treatment.

## Data Availability

The original contributions presented in the study are included in the article/supplementary material. Further inquiries can be directed to the corresponding author.
